# Do different response formats affect how test takers approach a clinical reasoning task? An experimental study on antecedents of diagnostic accuracy using a constructed response and a selected response format

**DOI:** 10.1007/s10459-021-10052-z

**Published:** 2021-05-11

**Authors:** Stefan K. Schauber, Stefanie C. Hautz, Juliane E. Kämmer, Fabian Stroben, Wolf E. Hautz

**Affiliations:** 1 Centre for Health Sciences Education, Faculty of Medicine, University of Oslo, Postboks 1161 Blindern, 0318 Oslo, Norway; 2 Department of Emergency Medicine, Inselspital University Hospital, University of Berne, 3010 Freiburgstrasse, Berne, Switzerland; 3 Center for Adaptive Rationality (ARC), Max Planck Institute for Human Development, Lentzeallee 94, 14195 Berlin, Germany; 4 AG Progress Test Medizin, Charité Medical School Berlin, Hannoversche Straße 19, 10115 Berlin, Germany; 5 Office of the Vice Dean for Teaching and Learning, Charité Universitätsmedizin, Berlin, Germany; 6 Centre for Educational Measurement (CEMO), University of Oslo, Postboks 1161 Blindern, 0318 Oslo, Norway

**Keywords:** Clinical reasoning, Response format, Cognitive reflection, Processing fluency

## Abstract

The use of response formats in assessments of medical knowledge and clinical reasoning continues to be the focus of both research and debate. In this article, we report on an experimental study in which we address the question of how much list-type selected response formats and short-essay type constructed response formats are related to differences in how test takers approach clinical reasoning tasks. The design of this study was informed by a framework developed within cognitive psychology which stresses the importance of the interplay between two components of reasoning—self-monitoring and response inhibition—while solving a task or case. The results presented support the argument that different response formats are related to different processing behavior. Importantly, the pattern of how different factors are related to a correct response in both situations seem to be well in line with contemporary accounts of reasoning. Consequently, we argue that when designing assessments of clinical reasoning, it is crucial to tap into the different facets of this complex and important medical process.

## Introduction

A crucial decision when designing an educational assessment is the selection of the format in which test takers give their response to the tasks or problems posed. Both research on assessment in medical education (Desjardins et al., 2014; Heemskerk et al., 2008; Huwendiek et al., 2017; Norman et al., 1996; Sam et al., 2018; Schuwirth et al., 1996) and the broader literature on educational and psychological testing (Bleske-Rechek et al., 2007; DeMars, 1998, 2000; Hickson et al., 2012; Lukhele et al., 1994; Rodriguez, 2003) have repeatedly focused on the question of in how far different item response formats evoke differences in task processing behavior.

Scholars within the field of educational testing typically draw a distinction between constructed and selected response formats. Selected response (SR) formats include all types of questions where test takers have to pick the correct option(s) out of a list. These are, for instance, multiple-choice, true/false, or multiple response questions. Constructed response (CR) formats, on the other hand, are questions or tasks where test takers have to generate the answer on their own. For example, this is the case in any essay or simply when test takers have to write in a single term (e.g. a diagnosis). Indeed, there is a common suspicion that these two broader classes of response formats evoke fundamentally different cognitive processes. Recognizing the correct option is perceived as being very different from generating the answer (Lissitz et al., 2012; Martinez, 1999; Ozuru et al., 2013). The main concern here is that the use of selected response formats would compromise the validity of assessments. If true, this would have important implications for both the design of assessments in medical education and research on clinical reasoning. However, empirical data to support these claims are limited (Hift, 2014; Norman et al., 1996).

On a pragmatic level, research shows that variations in response formats hardly affect actual assessment outcomes. That is, across disciplines, studies typically find high correlations between performances on tests using SR and CR formats (Martinez, 1999; Rodriguez, 2003). For example, in a recent article, Desjardins et al. (2014), found a correlation of r = 0.83 between performances on SR and CR tests using identical item-stems (i.e., identical clinical cases) in both conditions. At the same time, studies also report a consistent difference—selected response items are typically easier to answer. Compared to constructed response formats, test takers answer correctly more often when a SR format is used even if the problem posed is identical (Norman et al., 1996; Sam et al., 2018; Schuwirth et al., 1996). However, research also shows that using a SR format can make a task more difficult. This occurs when the options contain a highly attractive, but incorrect answer (Desjardins et al. 2014; Schuwirth et al., 1996). Importantly, these findings clearly highlight that test takers indeed make use of the options presented in a selected response format—even though the same options might, at times, be misleading.

Obviously, the possibility to uncover differences and similarities necessarily depends on the framework used to conceptualize response behavior or processes. Typically, studies conducted within medical education have been based on dual-process models. For instance, one popular framework differentiates between inductive and deductive reasoning (Elstein et al., 1978; Patel et al., 1993). Hence, the according studies use think-aloud techniques to reveal this type of reasoning (Heemskerk et al. 2008). Others focus on the reasoning as either intuitive/fast or elaborate/slow (Monteiro & Norman, 2013); in the according studies, analysis of response times plays a crucial role (Monteiro et al. 2015). In this paper, we aim at offering a new perspective on response behavior in a clinical reasoning scenario, which, in turn, introduces a new methodological approach, too.

In this study, we use a more recent framework that focusses on understanding the origins of errors in reasoning (De Neys, 2013, 2014; De Neys & Bonnefon, 2013; De Neys & Glumicic, 2008). DeNeys’ approach is rooted in dual process theory as it assumes that a response can be rather intuitive or more elaborate. The authors postulate that errors can originate from three elementary components of reasoning: storage, self-monitoring, and response inhibition. The first component, storage, means that reasoners answer incorrectly because they simply might not know or know wrong. Incorrect knowledge or misconceptions are, however, typically acquired long before an actual task is processed. The second crucial component is self-monitoring, which occurs while working on a problem. For instance, the feeling of being confident can determine the course of how a participant engages in solving a task or problem (Thompson et al., 2011). Critically, the third and final component is the ability to inhibit an intuitive response. Such inhibition is regarded as a key element in order to be able to adapt the actual reasoning process, that is, to switch from an intuitive response to a more elaborate one. While this framework resembles current thinking in research on clinical reasoning (e.g., Norman & Eva, 2010), there are a number of critical differences and additions, especially in regard to how self-monitoring and inhibition are understood.

The first difference is related to how various measures self-monitoring are subsumed in one single indicator of task-fluency. Task fluency is a reasoning person’s experience of processing a problem (Benjamin et al., 1998; Oppenheimer, 2008). This perception is formed by three indicators: The appraisal of something being difficult, which leads to increased time-on-task, which then results in a judgment of low confidence (Alter & Oppenheimer, 2009; Dunlosky, & Thiede, 2013; Hertwig et al., 2008; Koriat, 2012; Kornell et al., 2011). These three aspects, taken together, are indicators of task fluency, which, in turn, is a crucial trigger that can alter the reasoning process. For instance, reasoners might engage in more elaborate reasoning if they perceive low task fluency (Alter et al., 2007). This idea has its counterpart in research on overconfidence in diagnostic reasoning. Here too, the ability to appraise one’s own lack of knowledge or expertise is assumed to be crucial for avoiding––sometimes serious––errors (Berner & Graber, 2008). In summary, while there are many similarities to the concept of self-monitoring, task fluency allows for integrating different measures into a theoretically informed single indicator.

Importantly, Second, the approach by DeNeys and colleagues assumes that inhibiting an initially attractive, intuitive response is a crucial capacity in any reasoning scenario (Pennycook et al., 2015; Thompson et al., 2013; Toplak et al., 2014). Much of the thinking on such inhibitory processes is related to a paradigm used in the Cognitive Reflection Test (Fredrick, 2005). The starting point in these studies is to create tasks or problems that tend to trigger an immediate, but incorrect response. In order to solve a problem correctly, participants have to inhibit and overwrite this spontaneous response. For instance, one of the tasks in the cognitive reflection test goes as follows: *A bat and a ball cost $1.10 in total. The bat costs $1.00 more than the ball. How much does the ball cost?* The prepotent, incorrect, response in this case is “10 cents”, and, indeed, many participants give this intuitive response (Toplak et al., 2011). In order to answer correctly to such questions (assuming that they do not already know the solution), participants have to realize that the intuitive solution is incorrect, and overwrite their first response.

Since its first publication, the Cognitive Reflection Test (CRT) has been used in a wide range of studies, and substantial correlations to a number of indicators of faulty or biased reasoning have been found (Białek & Sawicki, 2018). In its original conception, the validity of the claim that this test indeed is an indicator of the ability to inhibit spontaneous responses has been mostly justified on a theoretical basis by the design of the tasks or questions. More recently, studies have focused on tracing participants’ response behavior when working through problems where they have to inhibit a prepotent answer (Toplak et al., 2011; Travers et al., 2016).

Indeed, the ability to inhibit and reflect on a prepotent answer may play a crucial, but differential, role in any testing scenario. In the particular context discussed here, SR and CR formats presumably differ with respect to the demands they set for test takes. Two strands of research support this expectation. First, previous studies found that more intuitive response behavior is more likely observed in SR format conditions (Heemskerk et al., 2008). Second, studies by Kostopoulou et al. (2012, 2015, 2016) found that reconsidering an initial hypothesis improves accuracy. In her studies, presenting a list of likely diagnoses was an especially effective intervention. Furthermore, Mamede and colleagues repeatedly demonstrated that deliberately reflection on difficult cases improves diagnostic accuracy, too (Mamede et al, 2012, 2020). Taken together, these findings suggest that, firstly, responses in SR conditions are more likely to be given intuitively but also that, secondly, reconsidering a spontaneous answer can improve accuracy.

The research reviewed above highlights an obvious contradiction. On the one hand, studies largely find that the scores obtained from assessments using one of the two formats are highly correlated—suggesting that the formats are largely exchangeable. At the same, both theoretical approaches and empirical evidence support the stance that different response formats trigger differences in cognitive demands and response behavior. Indeed, very different processes might still lead to the same scores.

Against this backdrop, we aim at addressing the question of why scores differ between CR and SR formats from a new perspective. We use DeNeys’ account of three elementary factors of reasoning to formulate expectations on where in the response process such differences actually occur. This account introduces a new perspective on investigating the effects of response formats in clinical reasoning tasks. It critically adds in two distinct ways theoretically and methodologically. First, it opens up the perspective that different response formats can be understood as setting different demands to the test taker, but, crucially, neither of them is inherently “more” or “less” valid. Second, this perspective builds on the conception of fast/slow thinking, but also goes beyond it in an important way. By stressing the importance of inhibition and, consecutively, reflection in intuitive response behavior, this framework accounts for some of the criticism dual process models are faced with (Evans, 2008; Kruglanski & Gigerenzer, 2011). Hence, the current study adds to the literature in two critical ways:

- On a theoretical level, we introduce a contemporary framework from cognitive psychology to health science education in order to reframe the issue on differences in response formats.
- On an empirical level, we formulate and investigate research questions on the role of these components in processing clinical reasoning tasks.

We conducted an experimental study in which we investigate the effect of a selected response format versus a constructed response format on response behavior in a clinical reasoning scenario. In our study, we assume that case-specific knowledge was independent of the testing condition, as we conducted a randomized study. Furthermore, we address two main research questions. First, we expect that differences in perceived fluency are related to differences in accuracy across cases. Second, we expect that scores on the CRT—as an indicator of the ability to inhibit a response—are differently related to accuracy across the two experimental conditions (CR, SR). Since one of the concerns frequently raised in regard to SR questions is that testees could simply guess the correct answer, we formulate a fourth research question. We speculate that reported guessing is related to low perceived fluency and consequently to lower chances of success.

## Methods

### Participants

The study was conducted at Charité–Universitätsmedizin Berlin. We invited all 350 medical students in their 4th academic year via email and through Facebook postings to participate in a “study of factors affecting decision making in emergency medicine”. Participation was voluntary and the institutional review board of Charité—Universitätsmedizin granted the study its approval (EA4/096/16). The first 60 replying students were invited to participate in the study. A total number of N = 54 students (67% females) took part. On average, participants were M = 24.6 (SD = 3.38) years old.

### Procedure

Upon arrival, participants were randomly assigned to one of two experimental conditions – CR (N = 27) or SR (N = 27). Participants were then informed about the study procedure and signed the consent form. After filling in a questionnaire on general demographic information, participants received a demonstration of how to work on the clinical cases. In total, one training case plus six clinical cases were administered, the latter were presented in random order. After participants had completed the cases, the Cognitive Reflection Test (Frederick, 2005) was administered. The complete session lasted about one hour, for which participants were compensated with €20 ($22 at that time).

### Clinical cases

We administered the ASCLIRE (Kunina-Habenicht et al., 2015) assessment, which consists of six clinical cases plus one trial case. Each case presents a patient with shortness of breath. All cases depict common causes of acute and sub-acute dyspnea. Participants were instructed to take all diagnostic tests they deemed relevant but no more than that. A total number of 30 diagnostic tests are available to choose from. Clicking on a test elicited the finding in the form of a text (e.g., pulse rate), an image (e.g., ECG, chest X-ray), or audio (e.g., heart sounds, history). Where feasible, these findings require the participants’ interpretation (e.g., ECG, heart sounds). Some findings (e.g., ultrasound exams, CT scans) are available only as radiologists’ textual report. Participants were free to choose any type, order and number of diagnostic tests they wanted to see or listen to, and repeated acquisition was allowed.

Participants were instructed to diagnose the patient as fast as possible without sacrificing accuracy. Importantly, students could decide to end the information-gathering phase and move on to giving their diagnosis. Once they decided to move on, they were no longer able to obtain clinical tests for the case. This procedure allowed for separating the time students took for obtaining information and processing the case form the time they needed to enter their diagnosis. After submitting their diagnosis and before proceeding to the next case, all participants were asked to evaluate each case with regard to its difficulty, whether or not they were guessing, and to what extent they were confident in the correctness of the diagnosis.

In the selected response condition, participants were free to choose a diagnosis by selecting one out of a list of 20. No return to the diagnostic tests was possible at this point. The list of possible diagnoses was the same for all cases and ordered alphabetically. In the constructed response condition, participants entered their diagnosis into a free text form after they finished processing the case. No return to the diagnostic tests was possible at this point. Three board certified emergency physicians with each at least 10 years of professional experience independently evaluated each CR response, blinded towards which examinee provided it. The final accuracy score was then derived from the majority of raters.

In the study by Kunina-Habenicht et al. (2015), a Cronbach’s Alpha of Alpha = 0.48 across the six cases was reported. The authors provide further evidence for the validity of the ASCLIRE framework in the said article.

## Measures

### Accuracy

The main outcome measure was whether or not students found the correct diagnoses to the presented cases. Accuracy of the selected diagnoses was treated as a dichotomous measure (correct or incorrect).

### Conflict detection and indicators of task fluency

The meta-cognitive measures obtained were confidence and perceived case difficulty. Furthermore, the time on a case was recorded in seconds. Confidence in the correctness of the diagnosis was rated on a percent-scale from 0% (*no confidence*) to 100% (*highest confidence)* in 10% increments. Perceived case difficulty was evaluated on a 5-point rating scale from 1 (*very easy*) to 5 (*very difficult*).

### Combined fluency score

In order to build a combined fluency score, self-reports on confidence and difficulty as well as time spent on case were standardized within each participant across cases with a mean of M_*within*_ = 0 and a standard deviation of SD_*within*_ = 1 (i.e.,within-person centred). We then multiplicated the z-standardized time-on-case and difficulty ratings by minus 1, thus reversing these two measures. In this way, the three variables had the same interpretation with regard to the fluency measure: Higher values on the three variables signified higher fluency. After centering and reversing, an average score was calculated using the three variables within every case. As a result, we obtained one fluency score for each person on each case (where higher scores indicate higher fluency). Hence, these scores carry information on the relative fluency experienced between cases and within each person.

### Cognitive reflection test

We administered a German version of the three items cognitive reflection test (Frederick, 2005) after students completed the six clinical cases. The score on the CRT was calculated as the number of correct responses on the three-item test. Reliability was determined by means of Cronbach’s Alpha.

### Guessing

Participants reported guessing per case dichotomously. After submitting a diagnosis, they received the following prompt: “Thank you for your diagnosis. Did you guess it? Yes/no”.

## Analytic procedure

Generalized linear mixed models (GLMMs) were used to analyze between-group differences in chances to solve a case correctly. The models used had the general form of:

$$logit\left( {P_{ij} } \right) = \gamma_{0} + \mathop \sum \limits_{h = 1}^{r} \gamma_{h} x_{hij} + S_{0i} + C_{0j}$$

where $P_{ij}$ is the odds ratio for subject *i* giving a correct response to case *j*,$\gamma_{0}$ indicates the intercept, and $S_{0i}$ and $C_{0j}$ represent the random intercepts for subjects and cases, both following a normal distribution with a mean of 0 and standard deviations of $\tau_{00}$ and $\omega_{00}$, respectively. As usual, the residual term in a logistic model is fixed to $\frac{{\pi^{2} }}{3}$ (≈3.29) and hence remains constant across all models. The sum $\mathop \sum \limits_{h = 1}^{r} \gamma_{h} x_{hij}$ represents the X_*(h*=1, …, *r*)_ predictors while $\gamma_{h}$ represents the according fixed effect. This means that $x_{hij}$ represents the value of subject *i* on case *j* for predictor X_*h*_. For example, $x_{4,3,2}$ would signify subject 3´s response on case 2 to the question of whether she was guessing the diagnosis on that case or not (“0” or “1”). The *r* = 4 predictors (response format, CRT, task fluency, and reported guessing) were entered successively meaning that, in total, 4 increasingly complex models were estimated.

We calculated the explained variance at the level of the random effects by calculating the proportional reduction of variance at the given level in relation to a Null Model (i.e., a model including an intercept only). Details on this procedure can be found in Snijders and Bosker (2011). The threshold for statistical significance was set at *p* = 5%. The package lme4 (Bates et al., 2015) within the R Language and Environment for Statistical Computing (R Core Team, 2018) was used to estimate the models.

## Results

### Descriptive statistics

Please refer to Table 1 for descriptive statistics for both groups and all cases. Case 4 (‘pneumonia’) was the easiest case, diagnosed correctly by 93% of the participants in the SR group and by 92% in the CR group. Across the two conditions, the average Pearson correlation of accuracy across all six cases across the experimental conditions was 0.09, and ranged between a minimum of – 0.05 (case two and case six) and a maximum of 0.28 between case 3 (pulmonary edema) and case 6 (intoxication). For the Cognitive Reflection Test, Cronbach’s Alpha was α = 0.81.

**Table 1:** Descriptive statistics for the six presentations per group

Clinical case	Condition	Diagnostic accuracy	Reported Guessing	Confidence	Experienced difficulty	Time on case (in seconds)	Time for submitting response (in seconds)	Fluency (z-scores)
1: instable ventricular tachycardia	SR	44%	26%	62.96 (18.77)	3.48 (0.94)	158.95 (82.98)	26.64 (15.69)	– 0.06 (0.65)
	CR	23%	15%	60.38 (23.91)	3.58 (0.95)	160.22 (94.22)	16.83 (26.04)	
2: chronic obstructive pulmonary disease	SR	93%	15%	76.67 (16.64)	2.52 (1.12)	125.09 (65.5)	9.97 (10.42)	0.34 (0.68)
	CR	77%	4%	66.54 (19.58)	3.23 (0.82)	171.06 (78.86)	21.35 (12.34)	
3: pulmonary edema	SR	52%	15%	64.44 (25.47)	3.41 (1.01)	168.01 (76.24)	29.77 (31.1)	– 0.23 (0.58)
	CR	31%	12%	54.23 (22.48)	3.88 (0.65)	249.53 (144.85)	17.88 (17.6)	
4: pneumonia	SR	93%	11%	78.15 (15.94)	2.59 (1.05)	124.32 (66.14)	15.62 (11.52)	0.41 (0.52)
	CR	92%	15%	66.15 (20.8)	2.85 (0.83)	156.6 (98.5)	13.98 (6.11)	
5: pulmonary artery embolism	SR	59%	22%	62.96 (27.29)	3.56 (1.22)	176.05 (105.64)	22.15 (23.8)	– 0.06 (0.71)
	CR	50%	15%	60.38 (24.41)	3.62 (0.98)	174.48 (82.1)	13.87 (15.48)	
6: intoxication	SR	56%	30%	57.04 (25.99)	3.7 (0.95)	176.74 (124.57)	23.45 (20.3)	– 0.39 (0.78)
	CR	42%	31%	46.92 (26.04)	3.96 (1.08)	214.14 (112.3)	25.92 (30.78)	

Mean for diagnostic accuracy, reported guessing (both in percent). Mean (SD) for confidence, experienced difficulty and time on case. Mean (SD) for the combined fluency scores on the case-level where higher scores indicate higher fluency

Table 1 gives descriptive statistics for the key measures in this study. For instance, the highest observed difference in guessing between groups were found for Case 2 (‘COPD’) and Case 1 (‘instable ventricular tachycardia’). For Case 2, 15% of the participants in the SR group (92% correct) reported guessing as opposed to 4% of the participants in the CR group (77%). For the most difficult case, Case 1, 26% of participants in the SR group and 15% in the CR group reported guessing. On average, guessing was more often reported in the SR group (20% vs. 15%). Furthermore, participants in the CR group indicated lower levels of confidence (*M*_*confCR*_ = 59.01, *SD*_*confCR*_ = 23.62; *M*_*confSR*_ = 67.04, *SD*_*confSR*_ = 23.16) and reported the cases as being more difficult (*M*_*diffCR*_= 3.52, *SD*_*diffCR*_= 0.96; *M*_*diffSR*_ = 3.21, *SD*_*diffSR*_ = 1.14). Importantly, these descriptive statistics serve an illustrative purpose and should be interpreted accordingly. Hence, no significance testing was conducted.

#### A combined measure of task fluency

Generally, the within-person centered indicators for time on task, confidence and perception of difficulty were correlated to each other with Pearson correlations of *r*_(time*_– _1, confidence)_ = 0.48, *r*_(time*−1, difficulty)_ = 0.62, and *r*_(difficulty*−1, confidence)_ = 0.74. All correlations were statistically significant with *p* < 0.001. In addition, we carried out a principal component analysis using Varimax rotation in the R package *psych* (Revelle, 2018). The results indicated that a common factor accounted for 74% of the variance in the observed variables. Furthermore, the correlation between the six case-level-averaged fluency measures and the six according case-level-averaged accuracies correlated with *r* = 0.87 (t = 3.49, df = 4, *p* = 0.03). Thus, the results were in line with our theoretical perspective and we summarized these measures into a single indicator of task fluency.

### Antecedents of accuracy (generalized linear mixed effects model)

In order to address our research objectives we fitted successively more complex models using generalized linear mixed effects models. Across models, the random effects structure was identical. In the following, we highlight the main findings; details for the models are given in Table 2.

**Table 2:** Results from the five different generalized mixed effects models

	Null Model		Model 1		Model 2		Model 3		Model 4	
	*OR*	*SE*	*OR*	*SE*	*OR*	*SE*	*OR*	*SE*	*OR*	*SE*
*Fixed parts*										
(Intercept)	1.79	0.93	2.59	1.41	0.90	0.55	0.97	0.57	0.46	0.65
Response format			0.47 *	0.15	0.50 *	0.15	0.47 *	0.31	1.84	0.67
Cognitive reflection					1.58 ***	0.22	1.65 ***	0.14	2.27 ***	0.21
Fluency							2.22 ***	0.21	2.21 ***	0.21
Guessing							0.51	0.38	0.50 *	0.38
Response format-cognitive reflection interaction									0.54 *	0.27
*Random parts*										
τ_00, person_	0.522		0.392		0.169		0.160		0.065	
ω_00, case_	1.428		1.432		1.455		1.085		1.083	
*Variance explained per level (in relation to null model)*										
Persons	–		25%		68%		66%		84%	
Cases	–		0%		– 2%		29%		29%	

*p < .05 , **p < ..01 , ***p < . .001

First, we only included the main effect for response format (i.e., the group effect) in the model. As expected, the result indicated that participants in the CR condition were less likely to give a correct diagnosis. The group effect alone explained 25% more of the variance on the between-person level as compared to the Null Model.

Second, we included both the main effect for the response format and CRT scores as fixed effects. Both were statistically significant predictors for giving a correct diagnosis and, combined, explained 43% additional variance on the between-person level (*OR* = 0.50, *p* = 0.011 and *OR* = 1.58, *p* < 0.001, respectively).

In a third step, we also included the fluency-related variables on the within-person level. This is, the person-specific variables task fluency and guessing that varied by cases. The results from this step indicate that, as expected, both a perception of low fluency and self-reported guessing were associated with decreased odds of giving a correct diagnosis (*OR* = 2.22, *p* < 0.001; *OR* = 0.51, *p* = 0.079, respectively). Both predictors combined explained 29% of the variance at the case level as compared to the null model.

Furthermore, we estimated a model introducing an interaction in order to investigate whether fluency was differently related to accuracy across the two conditions. The fluency-group interaction was not statistically significant (OR = 0.58; CI 0.27 – 1.25; *p* = 0.162). The newly introduced interaction did not add explained variance on any of the levels (not reported in Table 2).

The final, and most complex model introduced a response-format-CRT interaction. In this model, higher scores on the CRT were associated with more than doubling the chances of a correct diagnosis (*OR* = 2.27, *p* < 0.001). This, however, was only the case for the SR group; the effect for the CRT-Response-Type-Interaction in the CR group was *OR* = 0.54 (*p* = 0.026), thus canceling out the CRT-main-effect within this group. This model accounted for 84% of the variance on the between-person level and 29% of the variance on the case-level. Finally, both fluency and guessing were case-specific, that is, introducing these predictors to the model did only account for little variance on the between-person level (1%).

The significant interaction and the amount of variance explained associated with CRT scores pointed to a substantial difference in the correlation between diagnostic accuracy and performance on the CRT across the two experimental groups. Indeed, the rank correlation (Spearman) between CRT score and number of correct cases within the SR condition was r_(dx, crt)_ = 0.70 as compared to r_(dx, crt)_ =  – 0.07 within the CR group. We conducted a robustness check of this finding. Employing a bootstrap procedure, we found a 95% confidence interval for this correlation of 0.44 to 0.87 within the SR group. In the CR group, this interval was – 0.05 to 0.34.

### Predicting guessing

Finally, we fitted a GLMM in order to explain guessing on a given case. To do so, we basically exchanged two variables in the model; diagnostic accuracy became a predictor and guessing became the dependent variable. Furthermore, we included the person-centered fluency variable, and the CRT score to the predictors. The results indicated that guessing was less likely on cases where participants perceived high fluency (*OR* = 0.29; CI 0.17—0.49; *p* < 0.001). Neither the group-effect nor diagnostic accuracy, nor the CRT score showed a statistically significant relation (*OR* = 0.58, *p* = 0.35; *OR* = 0.67, *p* = 0.34; *OR* = 1.35, *p* = 0.28, respectively).

## Discussion

In this article, we report an experimental study in which participants were randomly assigned to complete six clinical cases on shortness of breath in a clinical problem-solving scenario using either constructed response or selected response answering format. Based on our review of the literature, we approached two main research questions—the relation between accuracy and task fluency on the one hand, and the relation between cognitive reflection and accuracy on the other hand.

Similar to previous studies we found that participants were able to monitor their performance on a case (Eva & Regehr, 2007, 2011; Kämmer et al., 2020). Furthermore, we did not find an effect of the response condition on the relation between the perception of task fluency and accuracy. However, our most critical finding was that CRT scores were related to higher accuracy in the SR condition, but not in the CR condition. In addition, we found support for our expectation that guessing was related to the perception of low fluency. Interestingly, we did not find an advantage of guessing: when participants reported guessing, they were more likely to be incorrect in their diagnosis.

Overall, we interpret these results as supporting the stance that different response formats evoke different response behavior and, at the same time, pose different demands on test takers. On the group level, some markers of task fluency varied between the two response formats—for instance, and as expected, it took more time to answer cases in the CR format. At the same time, it appeared that discrepancies in accuracy between the two conditions were strongly related to scores on the Cognitive Reflection Test (i.e., we found a significant CRT-response format interaction). Given the fact that we used an experimental, randomized design and with the theoretical framework introduced in mind, the main contender for explaining these differences in scores between groups is the differential role of response inhibition in both conditions. Interestingly, an interaction between response-format and task fluency was not significant. While this doesn’t allow for being interpreted as a null-effect, it still is interesting that the inhibition-response-format interaction was stronger than the interaction between fluency and response-format. We also want to mention that we did observe, as in many other studies in medical education, the phenomenon of ‘case specificity’—correlations of accuracy between cases were ranging between r = – 0.5 and r = 0.28. These were, indeed, in the range typically observed in clinical reasoning studies (Norman, 2008).

Studies focusing on the effect of response format in the assessment of clinical reasoning typically employ a theoretical framework rooted in dual-process theories (Monteiro & Norman, 2013), making a clear distinction between fast and slow reasoning and their relation to success or failure on a case (Heemskerk et al., 2008). Drawing on recent research in cognitive psychology, we aimed at extending this approach using the concept of response inhibition and cognitive reflection. This framework suggests that both being able to detect a conflict within an intuitive response and to inhibit this intuitive response is critical to successful, that is, accurate, reasoning.

In this study, we could only provide indirect empirical support for this conclusion because the measure for inhibition—the Cognitive Reflection Test—was a distal indicator of this faculty. Indeed, to date, most research on response inhibition relies on such indicators—observing inhibition in vivo and on the level of particular cases would clearly require a more fine-grained approach. Nevertheless, the framework applied here raises several questions that have interesting implications for the broader field of assessment of clinical reasoning. For instance, it raises the issue of how switching to ‘slow’ reasoning really can be induced experimentally. It is an open question whether a simple instruction to engage in deliberate reasoning really affects a certain reflection on the initial response. Specific designs of study tasks and conditions are, indeed, an option rarely endorsed in medical education research–but are quite common in research in cognitive psychology.

The current study has several limitations. First of all, although we conducted a randomized trial, the findings might still be the result of the specific composition of the groups analyzed here. Building on a sample of N = 54 participants and six cases, our study was still comparable in size to similar experimental studies in the context of research on clinical reasoning. In general, statistically, more extreme effects are likely to be found in such smaller samples. Therefore, the correlation patterns found here should be interpreted with caution. We did, however, perform a non-parametric bootstrap to investigate in which respect the association between diagnostic accuracy and CRT scores was influenced by the specific composition of the groups investigated here. The bootstrap suggests that the effect found might be at the higher end of possible effect sizes. However, the 95% bootstrap confidence intervals did not overlap, which suggests that the finding of differential correlation patterns across groups is reasonably robust.

Furthermore, we opted for a between-person design so that there were sufficient replications within person. Consequently, there is no possibility of investigating possible interactions between persons and response format. Finally, results obtained here might not be readily transferable to high stakes contexts, such as in licensing exams. Such scenarios are usually characterized by higher psychological strain or stress which may have additional effects not observable in this study.

Our findings have practical implications, too. We argue that there is not one type of response format that is generally `more valid` than another for the specific purpose of assessing clinical reasoning. Fenderson and colleagues claim that multiple choice tests tend to focus on trivia (Fenderson et al., 1997), a position, in our experience, frequently raised by lecturers and professionals in medical education. While this might be true in some contexts, it is, obviously, not the response format alone that triggers the type of reasoning processes but rather the task as a whole. Indeed, while scores are assumed to be largely comparable, the cognitive process preceding the actual answer might not. Hence, we agree with Desjardins and colleagues' (Desjardins et al., 2014) conclusion that the exclusive use of only one type of response format might have unfavorable effects and could ultimately impair the validity of a test or assessment. In this respect, we propose that our findings support the stance that the design of assessments in medical education should aim for using heterogeneous response formats.

In conclusion, our study introduced a new theoretical account of how to characterize differences in task processing and investigated how different response formats relate to different task processing behaviors. We argue that the findings presented here support the stance that different response formats are related to different processing behavior. Consequently, when designing assessments of clinical reasoning, it is crucial to tap into different facets of this complex and important medical process.
